# Adult-onset portosystemic encephalopathy caused by patent ductus venosus successfully treated with endovascular coil embolization: a rare case report

**DOI:** 10.1186/s42155-020-00118-1

**Published:** 2020-05-25

**Authors:** Hiromi Muranishi, Yasuo Komura

**Affiliations:** Department of Cardiovascular Intervention Radiology Center, Taijukai General Hospital Kaisei Hospital, 3-5-28 Muromachi, Sakaide-City, Kagawa 762-0007 Japan

**Keywords:** Symptomatic adult-onset patent ductus venosus, Portosystemic encephalopathy, Endovascular coil embolization

## Abstract

**Background:**

Patent ductus venosus (PDV) is a congenital shunt between the portal vein (PV) and inferior vena cava (IVC). However, there are few reports on symptomatic adult-onset PDV, and the appropriate management of this condition remains unknown. In particular, there are few reports on the use of endovascular therapy for the treatment of patients with symptomatic adult-onset PDV. However, the strategies, indications, long-term efficacy, and safety of this therapy remain poorly understood. Here we report a rare case of adult-onset PDV successfully treated via endovascular coil embolization using a retrievable IVC filter.

**Case presentation:**

A 35-year-old man with a clinical course of progressive general fatigue and ataxia for 3 months was diagnosed with depressive personality disorder in another hospital 2 months ago and then referred to our hospital for detailed examination and further treatment. Blood test results showed hyperammonemia, indicating hepatic encephalopathy. Contrast-enhanced multidetector computed tomography and transarterial portography revealed a portosystemic shunt that connected the left PV to IVC. Endovascular coil embolization was successfully performed after temporary balloon occlusion testing and the placement of a retrievable IVC filter. After the procedure, ammonia levels gradually reduced, and his symptoms improved without any postoperative complications. No clinical symptoms were observed at the 6-year clinical follow up.

**Conclusion:**

This report supports the findings of other studies and offers a less invasive therapeutic option, thereby aiding clinicians in making appropriate treatment decisions for these patients.

## Background

Several studies have described patent ductus venosus (PDV), a congenital shunt located between the portal vein (PV) and inferior vena cava (IVC). It is one of the causes of hepatic encephalopathy (HE) secondary to hyperammonemia in adults, but it is not well understood owing to its rarity and non-specific presentation; therefore, its diagnosis is often delayed. Consequently, these cases are often misdiagnosed as mental disorders (Saito et al. 2013; Watanabe 2000). Additionally, the optimal treatment management remains unclear. Symptomatic PDV management is classified into four types: low-protein diet therapy (Watanabe 2000), surgical management (Kamata et al. 2000; Perini et al. 2018; Kamimatsuse et al. 2010; Hara et al. 2013), liver transplantation, and endovascular therapy (EVT). To the best of our knowledge, there is limited information about adult-onset PDV in the literature; moreover, the results of the treatment methods used are unclear owing to the rarity of this condition. Furthermore, the number of case reports on the use of EVT for patients with PDV is limited (Saito et al. 2013; Schwartz et al. 1999; Marx et al. 2001; Shen et al. 2001; Chacko et al. 2016; Llanos et al. 2014; Cho et al. 2009; Araki et al. 2003; Maeda et al. 2009; Paudel and Hoffer 2015); of these reports, EVT was performed in only four adult patients. The short- and long-term efficacies and the safety of this procedure remain unclear. Hence, strategies for the use of transcatheter method to treat patients with PDV have not been established yet. Here we present an extremely rare case of symptomatic adult-onset PDV successfully treated via endovascular coil embolization.

## Case presentation

**Fig. 1:**
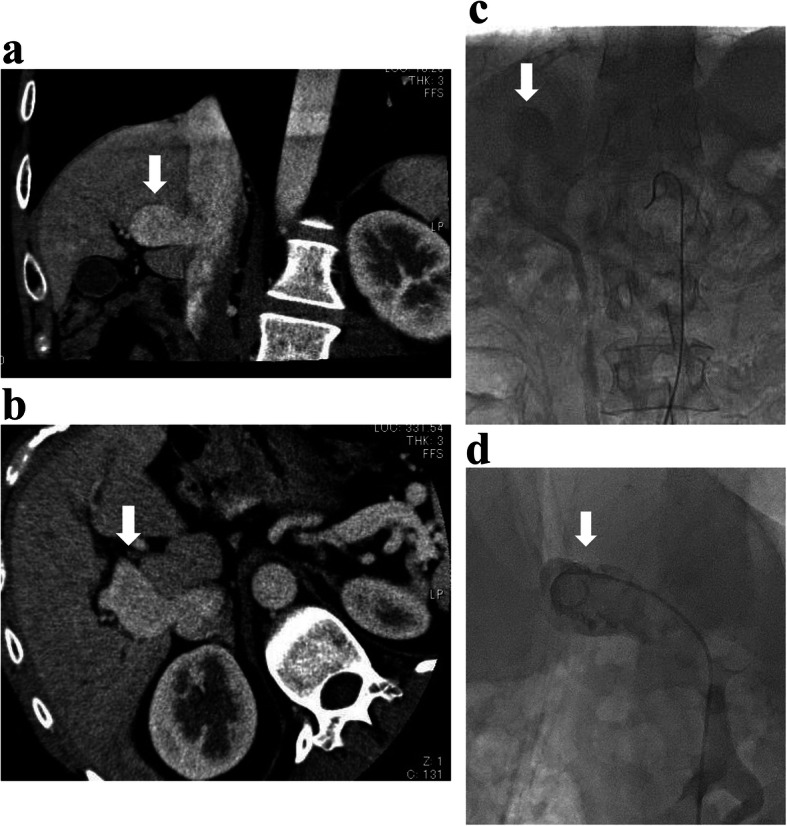
The findings of contrast-enhanced multidetector computed tomography (MDCT) and transcatheter angiogram. **a**, **b** Contrast-enhanced MDCT showing an abnormal venous connection between the left portal vein and the inferior vena cava (IVC; white arrows), suggestive of patent ductus venosus. **c**, **d** Elective superior mesenteric angiogram with delayed venous phase image and transfemoral venography showing the same findings as those of MDCT

We report the case of a 35-year-old man without any medical history who suffered from progressive general fatigue and transient mental confusion for 3 months. The patient was misdiagnosed with depressive personality disorder in another hospital, and the treatment was ineffective. Subsequently, the patient was referred to our hospital for further examination and treatment. Laboratory tests at our hospital revealed the presence of hyperammonemia (263 μg/dl), which suggested HE. Other laboratory data revealed no significant abnormalities, except low levels of serum albumin (3.0 g/dl) and a slightly elevated level of total bilirubin (1.4 mg/dl). Hepatitis B antigen and hepatitis C virus antibody were negative. Contrast-enhanced multidetector computed tomography (MDCT) suggested a portosystemic shunt that connected the left PV to the IVC (arrowhead in Fig. 1a, b).

Initially, conservative therapies including a low-protein diet were selected. However, they could not improve his symptoms owing to the presence of HE. A multidisciplinary team decided that surgical ligation might be associated with higher risks than endovascular coil embolization. Thus, informed consent was obtained from the patient and endovascular coil embolization under local anesthesia was planned. Before the procedure, elective superior mesenteric angiogram with delayed venous phase image and transfemoral venography were performed to confirm PDV diagnosis. Additionally, percutaneous liver biopsy was performed to discriminate pathological changes in the liver specimens (arrowhead in Fig. 1c, d). The findings of these examinations showed a portosystemic shunt that connected the left PV to the IVC and no evidences of hepatitis, cirrhosis, or veno-occlusive disease.

**Fig. 2:**
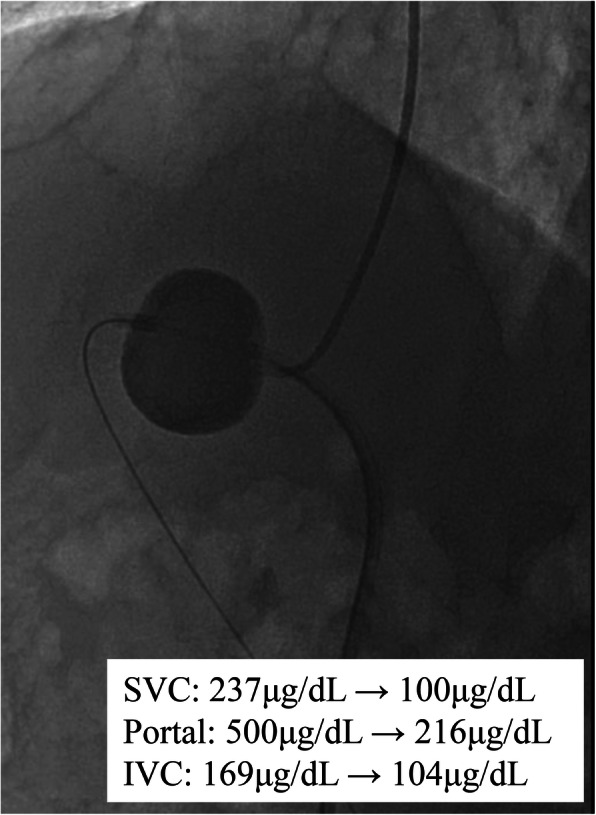
Temporary balloon occlusion testing was performed for 3 h. The laboratory test following temporary BOT shows decreased serum ammonia levels in SVC, portal vein, and IVC

**Fig. 3:**
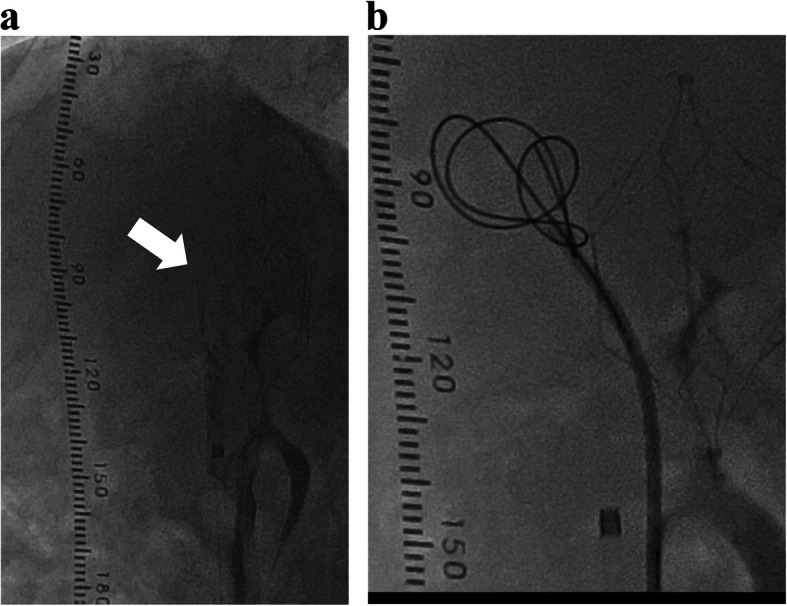
Coil embolization following placement of the IVC filter (white arrows)

**Fig. 4:**
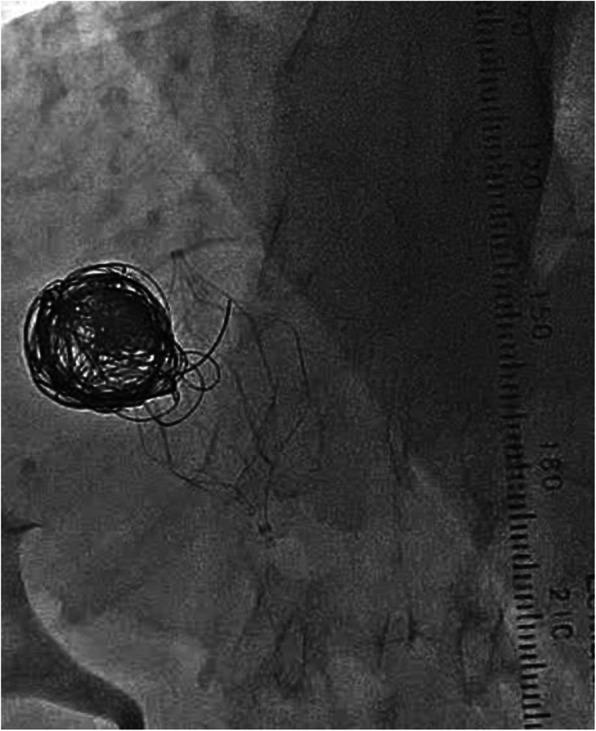
Coil embolization successfully performed using 18 coils

First, we planned to perform endovascular coil embolization using a 6-French catheter with a 20-mm-diameter balloon (Selecon MP catheter II: TERUMO Clinical Supply, Gifu, Japan). An 8-French sheath was inserted into the right jugular vein; following catheter insertion into the target vessel, the balloon was carefully inflated. However, the catheter was found to be floating and was easily pulled out. The aneurysmal dilatation diameter was found to be wider than that determined during the pre-procedure assessment. Therefore, we used a 7-French Swan-Ganz catheter (Edwards Lifescience, CA, USA) with a maximum balloon size diameter of 35 mm via the right common femoral vein (CFV), and it could occlude the target vessel completely. Additionally, complete occlusion was confirmed by catheter angiography via tip-hole of Swan-Ganz catheter and 5-French Judkins R. Temporary balloon occlusion testing (BOT) was performed for 3 h in intensive care unit to assess the changes in portal hemodynamics after complete embolization (Fig. 2). PV pressure increased from 10 to 30 mmHg after BOT. Laboratory results after 3 h showed a reduction in ammonia levels and no significant increase in transaminase. Moreover, abdominal ultrasonography showed no findings of congestive liver. Before coil embolization, a retrievable IVC filter (OptEase vena cava filter: Cordis Endovascular, A Johnson & Johnson company, Warren, NJ) had been placed to protect against coil migration at the connecting portion between the target vessel and IVC via the left CFV (arrowhead in Fig. 3a). A 6-French guiding catheter (Mach 1 MP: Boston Scientific, Natick, MA) was advanced into the target vessel via the right CFV. Coil embolization of the aneurysmal portion of the target vessel was accomplished using 18 coils: 6 interlocking detachable coils (Boston Scientific, Natick, MA) and 12 Trufill DCS Orbit Galaxy Complex Fill (Codman & Shurtleff, Inc. Raynham, MA) through a microcatheter (PROWLER SELECT Plus: Johnson & Johnson, Miami, FL, USA; Figs. 3b and 4). Immediately after coil embolization, his symptoms disappeared. However, it took 6 months for the serum ammonia levels to reach the normal range. Subsequently, the patient was free of the clinical symptoms and presented with no procedure-related complications during 6-year clinical follow up.

## Conclusions

Ductus venosus is naturally closed immediately after birth (Meyer and Lind 1966; Vade et al. 2001). PDV is a persisting congenital connection between the PV and IVC, and this extremely rare portosystemic shunt may be the cause of adult-onset portosystemic encephalopathy. To the best of our knowledge, there is limited information about adult-onset symptomatic PDV in the literature (Barsky et al. 1989; Barjon et al. 1972; Nakatsuji et al. 1991; Jacob et al. 1999; Nagano et al. 1999).

The treatment options for symptomatic PDV are as follows: conservative management with a low-protein diet, surgical management including liver transplantation, and endovascular management (Barsky et al. 1989; Franchi-Abella et al. 2010; Uchino et al. 1996; Orii et al. 1997). Patients with mild symptomatic adult-onset PDV may be treated via conservative management (Saito et al. 2013; Watanabe 2000; Araki et al. 2003). However, other interventional therapies can be indicated for patients with refractory symptoms, in addition to conservative therapy (Saito et al. 2013; Araki et al. 2003; Maeda et al. 2009). In recent years, the safety and efficacy of minimally invasive laparoscopic treatment for PDV has been reported (Perini et al. 2018; Takama et al. 2019; Hara et al. 2013). However, surgical management is generally more invasive owing to a complicated procedure and several potential risks, and the clinical results remain quite questionable (Barsky et al. 1989; Nakatsuji et al. 1991; Yamashita et al. 1982). In contrast, theoretically, EVT is less invasive. The first case of an adult patient who underwent coil embolization for PDV occlusion was reported in 2003 (Araki et al. 2003). There are extremely limited reports on the use of EVT for patients with symptomatic adult-onset PDV so far (Saito et al. 2013; Araki et al. 2003; Maeda et al. 2009). Several procedural strategies and techniques, including BOT before coil embolization and the use of various devices have been reported in all age groups (Saito et al. 2013; Araki et al. 2003; Maeda et al. 2009). However, the transcatheter method of treatment has not been established yet. In addition, the short and long-term efficacies and safety of this procedure remain unknown.

To the best of our knowledge, this is the first report of a patient who underwent coil embolization following retrievable IVC filter placement for PDV occlusion. Coil embolization has been performed along with balloon occlusion in previous studies to avoid coil migration (Araki et al. 2003; Maeda et al. 2009). We first attempted to occlude the target vessel with a balloon catheter; however, the balloon catheter was found to be floating and failed to occlude the exit of the target vessel completely because of a wider aneurysmal dilatation diameter than predicted. Thus, a 7-French Swan-Ganz catheter was used during BOT, and a retrievable IVC filter was placed to avoid coil migration during coil embolization.

In previous reports, more than half of the patients with PDV were complicated with absence or hypoplasia of the intrahepatic portal venous system and were indicated for liver transplantation or surgical ligation of the shunt after BOT (Wanless et al. 1985; Matsubara et al. 1996). Moreover, the PV pressure after BOT (under 22 mmHg) was reported to be a predictor of the postoperative risks (Kamimatsuse et al. 2010). In our case, percutaneous liver biopsy before procedure showed no significant abnormalities. Moreover, although the PV pressure after and 3-h temporary BOT was elevated to 30 mmHg, the patient was free of clinical symptoms with no procedure-related complications at the 6-year clinical follow up.

In conclusion, the findings of this case support those of other studies and indicate the efficacy and safety of endovascular coil embolization following retrievable IVC filter placement for the treatment of patients with symptomatic adult-onset PDV. This treatment approach may offer a less invasive therapeutic option.

## Data Availability

The datasets during and/or analyzed during the current case report are available from the corresponding author on reasonable request.

## Abbreviations

PDV: Patent ductus venosus
PV: Portal vein
IVC: Inferior vena cava
HE: Hepatic encephalopathy
EVT: Endovascular therapy
MDCT: Multi detector computed tomography
CFV: Common femoral vein
BOT: Balloon occlusion testing

## References

[CR1] Araki T, Konishi T, Yasuda S, Osada T, Araki T (2003). Embolization of the patent ductus venosus in an adult patient. Am J Roentgenol.

[CR2] Barjon P, Lamarque JL, Michel H, Fourcade J, Mimran A, Ginestie JF (1972). Persistent ductus venosus without portal hypertension in a young alcoholic man. Gut.

[CR3] Barsky MF, Rankin RN, Wall WJ, Ghent CN, Garcia B (1989). Patent ductus venosus: problems in assessment and management. Can J Surg.

[CR4] Chacko A, Kock C, Joshi JA, Mitchell L, Ahmad S (2016). Patent ductus venosus presenting with cholestatic jaundice in an infant with successful trans-catheter closure using a vascular plug device. Indian J Radiol Imaging.

[CR5] Cho YK, Chang NK, Ma JS (2009). Successful transcatheter closure of a large patent ductus venosus with the amplatzer vascular plug II. Pediatr Cardiol.

[CR6] Franchi-Abella S, Branchereau S, Lambert V, Fabre M, Steimberg C, Losay J, Riou JY, Pariente D, Gauthier F, Jacquemin E, Bernard O (2010). Complications of congenital portosystemic shunts in children: therapeutic options and outcomes. J Pediatr Gastroenterol Nutr.

[CR7] Hara Y, Sato Y, Yamamoto S, Oya H, Igarashi M, Abe S, Kokai H, Miura K, Suda T, Nomoto M, Aoyagi Y, Hatakeyama K (2013). Successful laparoscopic division of a patent ductus venosus: report of a case. Surg Today.

[CR8] Jacob S, Farr G, Vun DD, Takiff H, Mason A (1999). Hepatic manifestations of familial patent ductus venosus in adults. Gut.

[CR9] Kamata S, Kitayama Y, Usui N, Kuroda S, Nose K, Sawai T, Okada A (2000). Patent ductus venosus with a hypoplastic intrahepatic portal system presenting intrapulmonary shunt: a case treated with banding of the ductus venosus. J Pediatr Surg.

[CR10] Kamimatsuse A, Onitake Y, Kamei N, Tajima G, Sakura N, Sueda T, Hiyama E (2010). Surgical intervention for patent ductus venosus. Pediatr Surg Int.

[CR11] Llanos D, Armijo J, Bodas A, Vaquero E, Pedraja I, Arrazola J (2014). Transjugular closure of a patent ductus venosus in a symptomatic 14-year-old boy using a vascular plug. J Pediatr.

[CR12] Maeda M, Tazawa J, Mori K (2009). Transvenous embolization of patent ductus venosus in two adult cases. J Rural Med.

[CR13] Marx M, Huber WD, Crone J, Lammer J, Perneczky-Hintringer E, Heller S, Schlemmer M, Salzer-Muhar U (2001). Interventional stent implantation in a child with patent ductus venosus and pulmonary hypertension. Eur J Pediatr.

[CR14] Matsubara T, Sumazaki R, Saitoh H, Imai H, Nakayama J, Takita H (1996). Patent ductus venosus associated with tumor-like lesions of the liver in a young girl. J Pediatr Gastroenterol Nutr.

[CR15] Meyer W, Lind J (1966). The ductus venosus and the mechanism of its closure. Arch Dis Child.

[CR16] Nagano K, Hoshino H, Nishimura D, Katada N, Sano H, Kato K (1999). Patent ductus venosus. J Gastroenterol Hepatol.

[CR17] Nakatsuji Y, Kiyosawa K, Furuta K, Nakano Y, Suyama K, Sasaki Y, Yoshizawa K, Tanaka E, Sodeyama T, Furuta S, Nakanishi F, Imai Y, Horigome N, Kajikawa S, Iida F (1991). A case of hepatic encephalopathy and pulmonary hypertension due to intrahepatic portacaval shunt. Kanzo.

[CR18] Orii T, Ohkohchi N, Kato H, Doi H, Hirano T, Sekiguchi S, Akamatsu Y, Satomi S (1997). Liver transplantation for severe hypoxemia caused by patent ductus venosus. J Pediatr Surg.

[CR19] Paudel K, Hoffer EK (2015). Transhepatic embolization of congenital intrahepatic portosystemic venous shunts with associated aneurysms. Case Rep Med.

[CR20] Perini MV, Starkey GM, Goh SK, Riddiough GE, Christophi C (2018). Laparoscopic treatment of a patent ductus venosus and the use of indocyanine green to monitor perioperative hepatic function. J Surg case rep.

[CR21] Saito M, Seo Y, Yano Y, Momose K, Hirano H, Yoshida M, Azuma T (2013). Successful treatment using coil embolization of a symptomatic intrahepatic portosystemic venous shunt developing through a patent ductus venosus in a noncirrhotic adult. Intern Med.

[CR22] Schwartz YM, Berkowitz D, Lorber A (1999). Transvenous coil embolization of a patent ductus venosus in a 2-month-old child. Pediatrics.

[CR23] Shen B, Younossi ZM, Dolmatch B, Newman JS, Henderson JM, Ong JP, Gramlich T, Yamani M (2001). Patent ductus venosus in an adult presenting as pulmonary hypertension, right-sided heart failure, and portosystemic encephalopathy. Am J Med.

[CR24] Takama Y, Ueno T, Umeda S, Saka R, Tazuke Y, Okuyama H (2019). Laparoscopic ligation of a congenital extrahepatic portosystemic shunt for children with hyperammonemia: a single-institution experience. Surg Today.

[CR25] Uchino T, Endo F, Ikeda S, Shiraki K, Sera Y, Matsuda I (1996). Three brothers with progressive hepatic dysfunction and severe hepatic steatosis due to a patent ductus venosus. Gastroenterology.

[CR26] Vade A, Lim-Dunham J, Iqbal N (2001). Imaging of the ductus venosus in neonates: from patency to closure. J Ultrasound Med.

[CR27] Wanless IR, Lentz JS, Roberts EA (1985). Partial nodular transformation of liver in an adult with persistent ductus venosus. Review with hypothesis on pathogenesis. Arch Pathol Lab Med.

[CR28] Watanabe A (2000). Portal-systemic encephalopathy in non-cirrhotic patients: classification of clinical types, diagnosis and treatment. J Gastroenterol Hepatol.

[CR29] Yamashita S, Nakata K, Muro T, Furukawa R, Kusumoto Y, Munehisa T, Miyake S, Nagataki S, Ishii N, Koji T (1982). A case of hepatic encephalopathy due to diffuse intrahepatic Porto-systemic shunts. Nihon Naika Gakkai Zasshi J Jpn Soc Intern Med.

